# The limited prosocial effects of meditation: A systematic review and meta-analysis

**DOI:** 10.1038/s41598-018-20299-z

**Published:** 2018-02-05

**Authors:** Ute Kreplin, Miguel Farias, Inti A. Brazil

**Affiliations:** 1 School of Psychology, Massey University, Palmerston North, New Zealand; 2 Brain, Belief, & Behaviour Lab, Faculty of Health and Life Sciences, Coventry University, Coventry, England; 3 Donders Institute for Brain, Cognition and Behaviour, Radboud University, Nijmegen, The Netherlands; 4 Forensic Psychiatric Centre Pompestichting, Nijmegen, The Netherlands; 5 Collaborative Antwerp Psychiatric Research Institute, University of Antwerp, Antwerp, Belgium

## Abstract

Many individuals believe that meditation has the capacity to not only alleviate mental-illness but to improve prosociality. This article systematically reviewed and meta-analysed the effects of meditation interventions on prosociality in randomized controlled trials of healthy adults. Five types of social behaviours were identified: compassion, empathy, aggression, connectedness and prejudice. Although we found a moderate increase in prosociality following meditation, further analysis indicated that this effect was qualified by two factors: type of prosociality and methodological quality. Meditation interventions had an effect on compassion and empathy, but not on aggression, connectedness or prejudice. We further found that compassion levels only increased under two conditions: when the teacher in the meditation intervention was a co-author in the published study; and when the study employed a passive (waiting list) control group but not an active one. Contrary to popular beliefs that meditation will lead to prosocial changes, the results of this meta-analysis showed that the effects of meditation on prosociality were qualified by the type of prosociality and methodological quality of the study. We conclude by highlighting a number of biases and theoretical problems that need addressing to improve quality of research in this area.

## Introduction

**‘**If every eight-year-old in the world is taught meditation, the world will be without violence within one generation’ — this quote, attributed to the current Dalai Lama, and circulating on online forums, tweets and Facebook pages^1^, succinctly conveys the beliefs and expectations held by many about the powers of meditation. These vary considerably, from supernatural abilities (e.g., telepathy) to psychological states of peacefulness. Beliefs in the Western world about the powers of meditation became widely spread in the 1970s through the Transcendental Meditation movement^2^, a technique where one sits quietly and focuses on the mental repetition of a Sanskrit short word. The popularisation of Buddhist-based mindfulness meditation in the last two decades has further helped to promote the belief that meditation can be practiced as a faith-free method of inducing significant positive changes in consciousness^3^.

Buddhist mindfulness meditation was redefined as a non-religious technique of paying attention to the present moment with a non-judgemental awareness of inner and outer experiences that aim to create a state of ‘bare awareness’^4^. Its adaptation to a Western clinical context, originally aimed at chronic pain patients, paved the way for its popularisation through new mutations such as mindfulness based cognitive therapy (MBCT), which was developed to reduce relaxpse into depression^5^. As it became mainstream, mindfulness meditation was adapted to non-clinical contexts, including the corporate^6^ and the military worlds^7^, with the aim of increasing the well-being and work effectiveness of employees and soldiers. The utilization of meditation techniques by large corporations has created growing tensions within the wider community of individuals who practice and endorse its benefits. The more traditional practitioners and researchers advocate that mindfulness meditation without the ethical teachings can lead into the *wrong* kind of mindfulness^8^. An example of this would be that of the sniper who is fully mindful of his body, feelings, thoughts and intentions before pulling the trigger, which releases the bullet that will kill another human being^9^.

Although most of the popular claims and the scientific literature on the benefits of meditation have focused on isolated psychological and physical effects, there has always been a parallel interest in its inter-personal and collective effects. Dating back to the 1970s, Transcendental Meditation researchers published a number of studies reporting that this technique decreased aggression and violence at a societal level^10^. More recently, mindfulness and other Buddhism-derived meditation techniques (including compassion and loving kindness meditation) have been used to try to increase prosocial behaviours and feelings, such as compassion, social connection, and altruism^11–14^.

The studies on the prosocial effects of meditation have an obvious appeal. They not only help dispelling critiques of secular applications of meditation as self-centred or ethically misguided, but they support beliefs about the power of meditation – the power not only of transforming the individual but of changing society, as conveyed by the opening quote of this article. The possibility that meditation might improve prosocial behaviours, and reduce prejudice and aggression, brings with it the prospect of applications in a variety of contexts, including schools with high rates of conflict^15^ and in prisons^16^. It is conceivable that it may even find its use in social conflicts, such as mitigation of war and terrorism. Our primary aim in this article is to examine the extent to which the use of meditation-based techniques in healthy populations, outside of a religious context, might lead to improvements in prosociality. In other words, can meditation *per se* make the world a better — less aggressive and more compassionate —place? Our secondary aim was to test the influence of factors that may moderate this effect, such as the duration of the meditation.

As far as we are aware, this is the first systematic review and meta-analysis of the prosocial effects of meditation. Previous articles have reviewed the clinical benefits of compassion meditation (CM) and loving kindness meditation (LKM), both of which include a concern with stimulating positive other-centred emotions^17–19^. Importantly, there are differences between some types of meditation. While with mindfulness meditation one observes the flow of thoughts, feelings and sensations, in LKM and CM the aim is to focus on and elicit powerful positive feelings towards oneself, loved ones, and strangers. However, the literature is often unclear in which way compassion meditation is different from loving-kindness, though some authors suggest that the former focuses more particularly on the feeling of sharing suffering^19^. Prior studies employing these types of meditation were focussed on the clinical applications of meditation and, therefore, only partially looked at the prosocial effects of the interventions. Recognising that meditation may influence social behaviour, a recent meta-analysis of Buddhist meditation techniques considered a dimension representing ‘kindness and social domains’, alongside health, well-being and suffering^17^. It was concluded that the results for the clinical and social effects of the nine examined studies were encouraging, but inconsistent.

Here we sought to examine, through a systematic review and meta-analyses, if the power of meditation to elicit substantial improvements in various social variables (including compassion, connectedness, empathy, aggression, and prejudice) is empirically supported. Because of the lack of a theoretical agreement on the factors that underpin meditation, we also sought to assess potential moderators of its effects. Following the recent meta-analytical literature on meditation (e.g.,^20,21^), the role of expectation effects in meditation interventions^22^ and the variability introduced by different types of measures^23^, we considered as relevant moderators the duration of the intervention, the teacher’s involvement in the study, the type of control group, and the type of measures used to index social functioning. We did not have a priori expectations about the major categories of prosociality we would find. The search terms we used considered an array of prosocial variables (positive and negative), such as empathy and anger. The studies we selected, based on a set of criteria (see Literature Search section), revealed that the types of outcome measures most frequently used were: compassion, connectedness, empathy, aggression, and prejudice. We thus considered these five categories in our meta-analysis. Other conceptually interesting prosocial variables, such as forgiveness^24,25^, could not be considered in the meta-analysis due to incomplete reporting of statistical results. Because of successive criticisms about the poor methodological quality in meditation research^20,26^, we decided to only include randomised controlled trials (RCTs) that investigated the effects of mindfulness on social emotions (e.g., increased empathy, compassion and connectedness) and social behaviour (e.g., reduced aggression or prejudice) in healthy populations.

## Method

### Literature search and study selection

In May 2015 we searched PsycINFO, AMED, EMBASE, MEDLINE, PsychARTICLES, DirectSCIENCE, SCOPUS, and COCHRANE databases. We did not include year or language restriction. Because of criticisms concerning the methodological quality in meditation research^20,26^, we only included randomised controlled trials (RCTs) that investigated the effects of mindfulness on prosociality. Our search strategy included the words *meditation* or *mindfulness* in combination with any of the following terms: *empathy*, *relationship*, *connectedness*, *compassion*, *love*, *interpersonal*, *anger*, *social*, *altruism*, *outgroup*, *thankfulness*, *forgiveness*, *prosocial*. Our search strategy included only ‘meditation’ and ‘mindfulness’ to cover the whole breadth of meditation techniques. Note that the word ‘meditation’ is generally used as a composed noun, such as ‘lovingkindness meditation’, ‘transcendental meditation’ or ‘compassion meditation’. Mindfulness, however, is an exception as it is sometimes used as a single noun, which is why we have included it as a separate search term. Because our inclusion criteria focused on randomised controlled trials, we were unable to include a single study employing Transcendental Meditation. Although we found a number of studies on the prosocial effects of this technique, particularly on its supposed effect on reducing violence^10^, their methodology did not meet our inclusion criteria.

**Figure 1:**
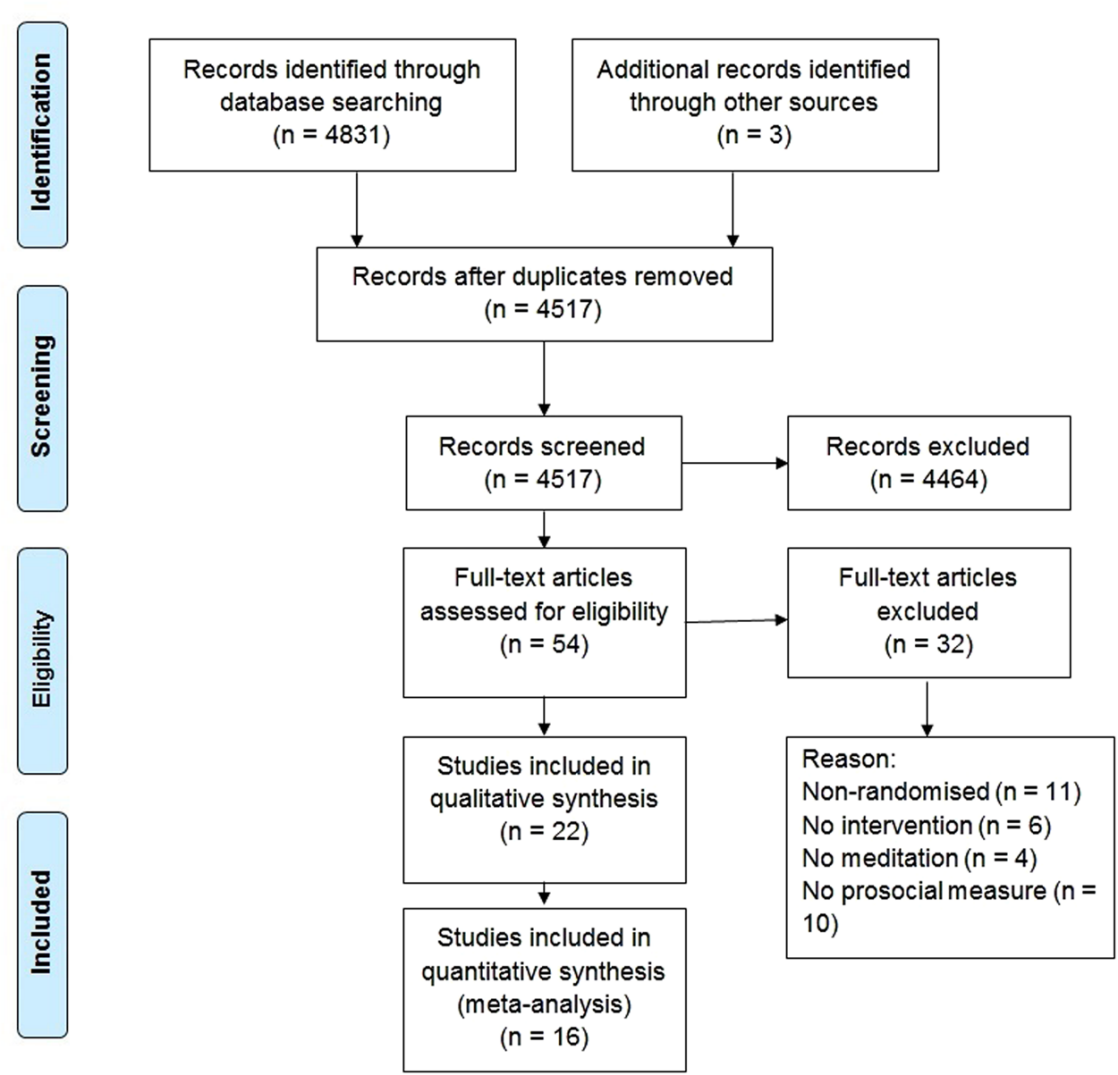
PRISMA 2009 Flow Diagram^64^.

Following the removal of duplicates, 4517 records were identified and screened (see Fig. 1). Two researchers independently read the titles and abstracts, excluding 4464 studies that did not meet the inclusion criteria (see Table 1), which left a total of 54 articles for full text analysis.

**Table 1:** Inclusion and Exclusion Criteria.

	Inclusion Criteria	Exclusion Criteria
Population	Healthy adults (>18)	Studies of children; studies using clinical populations
Intervention	Any structured meditation program including loving kindness meditation, mindfulness-based programs (e.g. MBSR, Zen and other mindfulness-based programs)	Meditation programs in which the meditation is not the foundation and most of the intervention (e.g. ACT). Any mind-body forms of exercise such as yoga, tai chi, and qi gong (chi kung); hypnosis; relaxation; pranayama
Study design	Randomized control trial (RCT)	Nonrandomised design and studies without a control group
Outcome variables	Prosocial variables (e.g. compassionate or empathic responding, forgiveness, helping behaviour, changes in anger and aggression)	Other variables

*Note:* ACT, acceptance and commitment therapy; MBCT, mindfulness-based cognitive therapy; MBSR, mindfulness-based stress reduction; RCT, randomised clinical trial.

The researchers read through the full studies and, following the exclusion criteria, rejected thirty studies (see Table 1 and Fig. 1). The twenty-two included articles (total *N* = 1685) reported studies that were randomised-control trials (RCT), used a meditation intervention and a passive or active control group, and had at least one outcome related to prosocial variables (e.g. Self-Compassion scale, Implicit Association Test of prejudice, empathic facial expression rating). We used a stringent definition of meditation as a form of focused attention to one or more elements, such as to one’s body, breath, conscious awareness, or to a particular word, thought or emotive state, which did not involve any physical activity. This excluded mind-body activities that sometimes involve meditation, such as yoga and Tai-Chi. We also only included interventions in which meditation was the predominant technique, which led to the exclusion of techniques such as Acceptance and Commitment Therapy.

### Data management

Studies were included in the meta-analyses if sufficient data were reported to allow calculation of effect sizes (i.e., pairwise comparison or correlations, sample size, descriptive statistics or *p* value for each test of interest). Studies frequently reported multiple assessments for a given measure (e.g., two scales for compassion). In such cases, effects were averaged across measures, except in cases where a separate entry was appropriate (e.g., a self-report and a behavioral measure). Sixteen of the 22 studies included in the systematic review also met the inclusion criteria for the meta-analysis. Social measures used in these sixteen studies were categorised into five types of prosocial feelings and behaviors: compassion, connectedness, empathy, aggression and prejudice. The latter two types are considered prosocial in a reversed way, as the studies under analysis are looking at their reduction. Categorisation was data driven.

The analyses were conducted following standard procedures (e.g.,^27,28^). Effect sizes were calculated as Person’s *r* using Meta-Calc. For all analyses, individual effect sizes were normalized through a Fisher’s *z* transformation and examined with random-effects models, as implemented in MetaWin^29^. Significance of the effects was determined with 95% Confidence Intervals (*CI*s), which should not contain the value 0. The *Q* statistic was used to assess heterogeneity^30^, and publication bias was estimated using Rosenthal’s fail-safe number with α = 0.05^31^. The fail-safe number provides an approximation of how many unpublished studies reporting null-results are required to render the effects of the meta-analyses insignificant.

### Moderator analyses

The studies assessed used a variety of prosocial measures and were also distinct in other aspects (e.g., type of measure and control group). Categorical moderator analyses were therefore conducted to assess whether any of these variables moderated the relationship between meditation and prosocial variables. The first moderator had five levels representing the type of prosociality to assess if the meditation intervention affected each type differently. Four further moderators with two levels were included (measure type, control group, teacher, and intervention duration; see Table 2 for details).

**Table 2:** Description and definition for categorical moderator variables.

Moderator Variable	Category	Definition
Prosocial Type	Compassion	Measures of pro-social behaviour
	Connectedness	
	Empathy	
	Aggression	
	Prejudice	
Measure Type	Questionnaire	Self-report questionnaires
	Behavioral	Behavioural tests such as IATs
Control Group	Active	Control groups that engaged in an activity (e.g. watching a video)
	Passive	Waiting-list control groups
Teacher	External/Audio	Where the teacher was not a named author in the article or instructions were given through audio recordings
	Author	Where the teacher was a named author in the article
Intervention	One-off	One off intervention sessions lasting from 3 min to 60 min
Duration	Multiple	Multiple intervention session lasting from 4 days to 3 months (6–8 weeks were most common)

### Quality of the studies

Methodological quality was assed using the Cochrane Collaboration’s risk-of-bias tool^32,33^ which included considering if the study: used random sequence generation; was advertised as a meditation intervention; controlled for confound variables (e.g. demand characteristics); included blinding of outcome assessment; and showed selective reporting of statistical results. We supplemented this with two further columns one being ‘intervention teacher’ to control for potential experimenter effects and demand characteristics, the other the type of control group the study had used (see Supplementary Table S2). When teacher information was not available in the study, authors were contacted via email and all, except one, answered.

Two researchers read and graded the twenty-two studies based on the Cochrane risk-of-bias tool. To further reduce bias from researchers undertaking the grading, we asked a third researcher who had no part in planning the review and meta-analysis, and is not a named author in this article, to also grade the methodological quality of the studies. The three researchers agreed on 72% of grades. Grades where disagreement arose were discussed until a consensus was reached. The quality grading resulted in three major outcomes: a score of 1 indicated *strong* confidence in the validity of the results and in its replicability; a score of 2 indicated *moderate* confidence in the validity of results, which may change with further studies; a score of 3 indicated *weak* confidence in the validity of the results and a greater likelihood that further studies might show contradictory evidence. A study would be graded as strong if it met six of the seven criteria described above, for example, if it showed no selective statistical reporting, used an active control group, employed a meditation teacher that was not a named co-author, it blinded outcome variables, and it controlled for confound variables. For a study to be graded as moderate it had to meet a minimum of three criteria. Studies meeting less than three of the quality criteria were graded as weak.

The datasets generated during and/or analysed during the current study are available from the corresponding author on reasonable request.

## Results

### Characteristics of selected studies

The earliest study included was published in 2004 but over two thirds of the studies (71%) were from 2010–2015, which shows a growing interest in assessing the prosocial effects of meditation. The techniques most commonly used were mindfulness-based interventions, LKM, and CM. The length of the interventions varied from 3 minutes to a 3-month meditation retreat, though more than a third of studies (39%) lasted for 8-weeks. Nine of the twenty-one selected studies included a control condition with an active task, which varied from watching a nature video to the use of other interventions, such as a time-management course. The remainder used wait list control groups. Supplementary Table S1 shows the characteristics of the selected studies.

### Quality of studies

The methodological quality of the studies was generally weak (61%), while one third (33%) was graded as moderate, and none had a grading of strong (see Supplementary Table S2). Only two studies assessed confounding factors, such as demand characteristics^34,35^, and only five reported the method of randomization^36–40^. All except one of the studies^41^, where the intervention was taught by a person, used a meditation teacher that was a named author in the study (information unavailable for two studies^24,25^). A further eight studies used written or recorded meditation instructions^13,35,39,42–46^.

### Results of the meta-analysis

The mean effect size for the overall analysis, in which the effect sizes were aggregated across all studies, was *r* = 0.26 (*CI* 0.18–0.34; see Table 3), showing that there is a moderate increase in prosociality following a meditation intervention. The non-significant heterogeneity *Q* statistic indicated that the overall sample was homogeneous and Rosenthal’s fail-safe number pointed out that 396 studies with null-results are needed to make the main effect found in this overall meta-analysis non-significant. As a rule of thumb, the fail-safe number is considered substantial when it exceeds 5*n* + 15, with *n* representing the number of studies in the meta-analysis. In the present study, the fail-safe number is 105 which is above the recommended threshold.

**Table 3:** Overall effect sizes and results for the categorical moderator analysis.

Category	All Measures				Compassion			
	ES	95% CI	Fail-Safe	Heterogeneity	ES	95% CI	Fail-Safe	Heterogeneity
Main effect	**0**.**26**	0.18, 0.34	396	*Q* 25.71	**0**.**36**	0.25, 0.48	181	Q 8.96
*Prosocial Type*								
Compassion	**0**.**37**	0.24, 0.49	580	Qb 15.32	N/A			
Connectedness	0.22	−0.05, 0.49		Qw 21.38				
Empathy	**0**.**44**	0.03, 0.84						
Aggression	0.11	−0.17, 0.48						
Prejudice	0.11	−0.09, 0.30						
*Measure Type*								
Questionnaire	**0**.**24**	0.13, 0.35	387	Qb 0.40	**0**.**43**	0.27, 0.59	221	Qb 3.004
Behavioural	**0**.**29**	0.15, 0.43		Qw 24.78	**0**.**27**	0.05, 0.49		Qw 7.96
*Control Group*								
Active	**0**.**27**	0.12, 0.43	380	Qb 0.08	0.37	−0.04, 0.78	160	Qb 0.006
Passive	**0**.**25**	0.14, 0.36		Qw 24.67	**0**.**36**	0.20, 0.79		Qw 7.92
*Teacher*								
External/Audio	**0**.**22**	0.09, 0.35	394	Qb 0.97	0.26	−0.07, 0.59	214	Qb 2.65
Author	**0**.**30**	0.18, 0.41		Qw 24.64	**0**.**42**	0.27, 0.57		Qw 7.97
*Intervention Duration*								
One session	**0**.**28**	0.07, 0.48	379	Qb 0.06	Fewer than two cases per category available			
Multiple sessions	**0**.**25**	0.16, 0.35		Qw 24.67				

*Note:* Significant results are highlighted in bold; Mean effect size (ES) reported as Pearson’s *r* with their corresponding 95% confidence interval (CI); Rosenthal’s fail-safe number, and *Q* heterogeneity statistic; Qb heterogeneity statistics for between-group and Qw for within-group effect size differences. The analysis contained 26 independent samples, exceeding the *N* of included studies because more than one outcome measure (e.g. compassion and empathy) was used in most studies.

The first set of moderator analyses, conducted using the full data set, showed a moderating effect for prosocial type only. The results indicated that compassion and empathy where affected by the meditation intervention (*r* = 0.37 and *r = *0.44, respectively). That is, meditation intervention had a positive effect on levels of empathy and compassion, relative to baseline. This was not the case for aggression, connectedness, and prejudice (see Table 3). Although the overall analysis indicated that there was homogeneity of effect sizes across studies, this was not the case once the total sample was divided into subgroups based on the different prosocial types, which showed high heterogeneity. This was probably caused by the considerable variety of outcome measures across studies. Note that the effect sizes were still homogeneous *within* each subgroup and that Rosenthal’s fail-safe number was high, thus supporting the robustness of the results.

We ran an additional set of moderator analyses for compassion data only, as the number of empathy studies was too low to conduct further analyses. The results indicated that compassion increased following a meditation intervention (*r* = 0.36, 95% *CI* 0.25, 0.48) and we found a moderation effect for type of control group and meditation teacher, but no other moderator (see Table 3). The moderator analysis for teacher indicated an increase in compassion for studies were the meditation teacher was a named author on the study (*r* = 0.42, 95% *CI* 0.27, 0.57), but not when the intervention was taught by someone not listed as an author or through paper/audio instructions (*r* = 0.26, 95% *CI* −0.07, 0.59). The moderator analysis for type of control group indicated that the results for levels of compassion were more varied when employing an active (*r* = 0.37, 95% *CI* −0.04, 0.78) *versus* a passive control group (*r* = 0.36, 95% *CI* 0.20, 0.79), which led to a non-significant effect when employing an active control group. All heterogeneity statistics for the moderators were non-significant and Rosenthal’s fail-safe numbers were large for all analyses (see Table 3 for details).

## General Discussion

We found that the effects of meditation interventions on prosociality were limited. The methodological quality of 61% of the studies was graded as weak. Although there was a moderate increase in prosociality when considering all studies, further analyses indicated that this effect was qualified by two factors: category of prosociality and methodological quality. Meditation interventions had an effect on the categories of compassion and empathy, but not on aggression, connectedness, or prejudice. The low number of empathy studies prevented a more detailed analysis of moderators. For the category of compassion, we found that methodological quality impacted the outcomes so that an increase from baseline to post-intervention was moderated by the use of a meditation teacher. Specifically, the moderation results showed that a significant increase in compassion only occurred if the intervention teacher was a co-author in the published study, but not when the intervention was delivered by other means (written/audio) or by a teacher that was not a co-author in the publication. The results for compassion were also moderated by the type of control group; specifically, the effect became non-significant when an active control group was used. Although the effect size remained similar for active and passive controls, the confidence intervals showed a much wider variation in the results for studies employing an active control group (−0.04, 0.78) indicating that some studies produced results where there were no changes in compassion, or there was a reduction from baseline to post-intervention. Overall, the weak methodological quality of the studies and the results for the moderator analysis indicates low confidence in the validity and replicability of the examined studies. The studies used a wide range of prosocial outcome measures, including self-report instruments, implicit association tasks, and behavioural measures. Not all of these had been previously validated, but the crux of the methodological shortcomings lies elsewhere, as the quality of studies analysis and moderator analysis reveals. Below we expand on these shortcomings and how they might be avoided in future studies.

On the whole, there was some evidence favouring the prosocial effects of meditation, but this was limited by various factors, including: (1) the finding that the initial results were moderated by the meditation effects on empathy and compassion alone; (2) that the effects on compassion were moderated by the type of control group used and the role of the teacher; (3) the weak methodological quality of the studies. Concerning point (1), it is intriguing that we found that the effects of meditation on empathy and compassion were significantly stronger than for the other types of prosociality. One explanation could be that the meditation interventions focused on the development of empathic and compassionate qualities. This is clearly the case for the majority of the compassion studies analysed, where the meditation training (LKM and CM) used statements that explicitly asked participants to focus on feelings such as ‘love’ and ‘kindness’ towards oneself and others. Also, these studies used as the major outcome measure a scale of Self-Compassion that assessed the same emotions that were elicited during the intervention^35,37,38,47^. Only one study attempted to extricate the affective element in the intervention by including two different types of meditation^11^. On the other hand, the studies on aggression, prejudice and connectedness tended to use a type of mindfulness-based meditation which did not directly mention qualities of reduced aggression, prejudice or increased connectedness.

Regarding point (2), that the effects of meditation on compassion were only significant when compared to passive control groups suggests that other forms of active interventions (like watching a nature video) might produce similar outcomes to meditation. Another meta-analysis has shown a similar pattern of non-significant or weak results concerning the effects of meditation on psychological stress and well-being when compared to active controls^20^. The second moderator we found is more controversial, and this seems to be a novel finding. In what way does the joint status of study co-author and meditation teacher affect the results of the compassion studies? At best this shows that a motivated meditation teacher will impact to a greater extent one’s students; at worse, it suggests that experimenter biases are introduced which affect the outcomes of the studies. These are just one kind of bias that are likely to be affecting studies in this area and which we review below (point 3), alongside offering potential solutions to overcoming them.

Our assessment of the quality of the studies identified several methodological weaknesses, which increase the likelihood that biases were introduced. First, despite Rosenthal’s^48^ well-known work on *experimenter biases* and the importance of using double blind designs in experimental psychology, meditation studies seldom try to avoid this particular bias. Recent work indicates that experimenter biases are not a thing of the past. When Doyen and colleagues^49^ attempted to replicate a previous experiment suggesting that priming participants with age-related stereotypes had an effect on walking speed^50^, they failed to find any significant results when using a double-blind procedure (prior studies were not blinded). They further showed that when making some experimenters believe that priming participants with age-related stereotypes would slow them down, this indeed had a significant effect, but only in those experimenters that were made to believe in the stereotypes. This example illustrates how experimenter beliefs can directly influence the outcome of a study.

In the context of the studies included in our review, authors provided the training in 48% of the studies. Only one study stated that the meditation instructor, although being part of the research team, had no part in the data analysis (Neff and Germer, personal communication) and another study engaged an external instructor who was not connected to the study in any other way^41^. Information about the intervention teacher could not be obtained for 10% of the studies and the remaining 42% used audio recordings or online instructions. In sum, for about half of the studies we reviewed, unintentional experimenter biases could have been introduced by researchers/teachers with a personal interest in the intervention (e.g., by giving preferential treatment or being particularly enthusiastic to participants in the experimental group).

But the prevalence of experimenter effects is only one side of the coin. The media portrayal of meditation as a cure for a range of mental health problems or to improve well-being^51^ is very likely to feedback into participants who will have a high expectation of the benefits of a meditation intervention. Despite potential to introduce unintentional *expectation bias* in participants, only one of the studies we examined controlled for expectation effects and this methodological concern is generally absent in the meditation literature. The exception was a study by Creswell and colleagues^22^, which included a four item scale assessing beliefs about the efficacy and relevance of the intervention (meditation versus analytical training) and found that the meditation group had substantially higher expectations of a positive effect for the intervention, even though participants were not explicitly told that they were engaging in meditation.

What is the solution to unintentional experimenter and expectation effects? Acknowledging them is a good starting point and supplementing this with short scales that try to measure participants’ expectations can only improve the validity of studies — for an example from the anxiety treatment literature, see the Credibility and Expectancy Questionnaire^52^. But more can be done. Importantly, larger gains can be made by introducing at least some aspect of blinding procedures in randomized controlled studies. Then, the challenge is to find suitable interventions that can function as active control conditions. An interesting solution was developed by Smith^53^, who developed a 71-page manual describing the rationale and benefits of a made-up meditation technique. He gave the manual to a research assistant, who was unaware that it was a placebo, and who then proceeded to give a lecture to participants in the control group about the merits of the technique (very much like in the experimental group that used Transcendental Meditation). When it came to the actual placebo technique, participants were instructed to sit quietly for 20 minutes twice per day in a dark room, and to think of anything they wanted. Although this was an innovative approach, we acknowledge that it may create other problems such as the elimination of intentional positive expectancy.

In sum, the negative impact of experimenter and expectation biases should not be overlooked in modern meditation studies, especially given the lack of double-blind designs in this field. Planning double-blind studies that use a placebo is possible and desirable in meditation research, particularly when dealing with the elicitation of positive emotions, such as compassion or empathy. Having a meditation teacher who knows nothing about the hypotheses of the study and has no part in designing, analysing and writing the results, would also reduce the likelihood of methodological biases.

Methodological flaws allow for many other biases, such as those concerning data analysis and reporting. Interpreting statistical results and choosing what to highlight is challenging. Kaptchuk^54^ has summarised a number of potential interpretative biases that have become widespread in science reporting, including a *confirmation bias*, where one tends to evaluate evidence that supports one’s beliefs more favourably than evidence that challenges it.

A confirmation bias was particularly prevalent in the studies we reviewed in the form of an over-reporting of ‘marginally significant’ results. In addition to statistically significant results (p < 0.05), 48% of studies reported marginally significant results (p > 0.05), which varied considerably — *p*-values ranged from 0.06 to 0.14. Further, the majority of studies failed to urge caution in the interpretation of these marginally significant results and, in some cases, discussed them *a par* with other statistically significant effects. This over-reliance on marginally significant results to generate theoretical interpretations naturally increases the chances of a Type I error^55^. Just to illustrate this bias, let us take an example from one of the studies we reviewed and meta-analysed. On p. 461, the authors reported a marginally significant difference (p = 0.069) in favour of the meditation intervention relative to the control group. However, on the following page, when the authors reported a different set of results that did not favour the meditation intervention they claimed the exact same p-level as *non-significant*: “The results confirmed our hypotheses for intergroup anxiety. Contrast 1 was not significant, *t*(75) = 1.85, *p* = 0.069” (p. 462,^44^).

Another potential instance of a confirmation bias we identified was the inconsistency of reported results in the way that meditation intervention effects were assessed. Some studies reported within-subjects effects from pre- to post-test, whereas others reported changes from post-testing to follow up, yet others only compared between-subject effects at post-test. It is unclear what exactly underpins this inconsistency, but it is likely to be the result of a bias to report significant results and neglect non-significant ones.

Potential suggestions to ameliorate confirmation biases include: a full disclosure of results, including all non-significant ones; a clear treatment of *p*-values as either significant or non-significant; and to run two different families of statistical tests, such as traditional null-hypothesis testing and non-parametric tests (e.g. bootstrapping), or Bayesian tests, and see if the findings converge. Meditation studies would benefit from being pre-registered to prevent ad-hoc analysis and reduce the experimental degrees of freedom during analysis. Finally, the presence of a confirmation bias has an impact on the interpretation of the results by biasing the generation of theoretical assumptions about their meaning.

The majority of studies we reviewed presented very tenuous and unclear justifications for why a meditation intervention ought to improve prosocial outcomes. The research literature tends to swiftly reference the health benefits of meditation and/or mention the alleged prosocial effects of meditation in the Buddhist tradition^36,38,41^. Further, this literature generally conveys the impression that Buddhism is particularly concerned with the promotion of prosociality and that meditation is the means to achieve it. This is a rather inaccurate understanding of a rich and plural religious tradition. Leading academics of South Asian religions have highlighted the Western misreading and reconstruction of Buddhism as a rational form of inquiry focused on meditation, which has been uncritically accepted by psychology researchers^56^. For example, such authors highlight that for most forms of Buddhism, it is not meditation but the study of sacred scriptures that is the most valued means to achieve deep personal transformation. Other scholars have also cast a critical light upon the definition of mindfulness as a process of paying attention, in the present moment and non-judgmentally^57^, regarding it as something different from what the Buddha scriptures describe – less than a form of attention or awareness to one’s thoughts, feelings and sensations, but rather a *reflection* upon the impermanence of all things, starting with one’s body^58^.

This is not the place to dwell upon the lack of agreement between psychology and the Eastern spiritual traditions on what meditation is and its precise role in effecting personal change. We simply wish to point out the conceptual mist which comes across in the reports we examined, either in the lack of an overall coherent theoretical framework, or even the lack of an attempt to theorise about how meditation works. Most of the reports focus on meditation as a tool that can be used for various purposes, such as the cure of social isolation^13^. Only rarely do studies try to look at underpinning mechanisms, such as the role of meditation in increasing empathic accuracy^43^ or in decreasing psychological stress^41^. However, the results either failed to show that the mechanism in question played any significant role or it only worked partially (reducing psychological distress when dealing with prejudice regarding homeless but not Black people)^41^.

The lack of a clear attempt to address underpinning mechanisms of meditation makes the literature more vulnerable to implicit magical beliefs about the power of Eastern contemplative techniques, even when adapted into medical and mental health settings. Previous systematic reviews and meta-analyses have voiced parallel concerns. For example, a recent review of LKM and CM found these techniques ill-defined and lacking standardised protocols^19^. Also, the majority of meta-analyses on the benefits of meditation acknowledge the pervasive methodological shortcomings of the studies analysed, but still suggest that such results are ‘encouraging’ or ‘promising’^20,21,26,59–62^. Unfortunately, such note of optimism is premature in what concerns the literature on the prosocial effects of meditation. We need new studies that take seriously the potential biases and methodological limitations we highlight above, as well as providing a clear theoretical grounding, including the role of potential psychological processes underpinning the prosocial effects of meditation.

### Conclusion: Can Meditation Make the World a Better Place?

All world religions promise that the world would change for the better if only people were to follow its rules and practices. The popularisation of meditation techniques in a secular format is offering the hope of a better self and a better world to many. In the early 1970s, Transcendental Meditation conveyed this message openly, announcing that the rising number of individuals practising this technique would lead to world peace in the short term^63^. Psychologists using mindfulness or other Buddhism-derived meditation techniques are now advancing similar ideas about the prosocial effects of meditation. In the foreword to the Mindfulness Initiative UK (2015) report launched at the British Parliament, Kabat-Zinn wrote of the profound potential of meditation to bring about societal changes. Despite these high hopes, our analysis suggests that meditating is likely to have a positive, but still relatively limited effect in making individuals feel or act in a substantially more socially connected, or less aggressive and prejudiced way. Compared to doing no new emotionally engaging activity, it might make one feel moderately more compassionate or empathic, but our findings suggest that these effects may be, at least in part, the result of methodological frailties, such as biases introduced by the meditation teacher, the type of control group used and the beliefs and expectations of participants about the power of meditation.

This, of course, does not invalidate Buddhist or other religions’ claims about the moral value and eventually life changing potential of its beliefs and practices. However, the adaptation of spiritual practices into the lab suffers from methodological weaknesses and is partly immersed in theoretical mist. Before good research can be conducted on the prosocial effects of meditation, these problems need to be addressed.

## Electronic supplementary material

Supplementary Tables
