# Supplementary material for: The limited prosocial effects of meditation: A systematic review and meta-analysis
Source: Sci Rep. 2018 Feb 5;8:2403. doi: 10.1038/s41598-018-20299-z (PMC5799363; doi:10.1038/s41598-018-20299-z)
Supplement: Supplementary file 1 — Supplementary Tables [file 41598_2018_20299_MOESM1_ESM.doc]

The limited prosocial effects of meditation: A systematic review and meta-analysis

Ute Kreplin*

School of Psychology Massey University, New Zealand Email: u.kreplin@massey.ac.nz

Miguel Farias* Brain, Belief, & Behaviour Lab, Faculty of Health and Life Sciences, Coventry University, England Email: miguel.farias@coventry.ac.uk

Inti A. Brazil -Donders Institute for Brain, Cognition and Behaviour, Radboud University, The Netherlands -Forensic Psychiatric Centre Pompestichting, Nijmegen, The Netherlands -Collaborative Antwerp Psychiatric Research Institute, University of Antwerp, Belgium - Brain, Belief, & Behaviour Lab, Faculty of Health and Life Sciences, Coventry University, UK Email: i.brazil@donders.ru.nl

*Joint first authors

| Supplementary Table 1 | | | | | |
| --- | --- | --- | --- | --- | --- |
| *Study Description* | |  |  |  |  |
| Source | Meditation Program | Program Duration | Outcomes | Population/Region of Study | N (final analysis) |
| †Arch et al., 2014 | Loving Kindness Meditation | 4 days | Self-Compassion Scale (+) | Undergraduate students/ US | 103 |
| †Bowen et al., 2012 | Mindfulness based intervention informed by functional analytic psychotherapy. Split into two groups, one with an interpersonal, one with an intrapersonal component. | 60 min | Interpersonal Group^1^: | Undergraduate students/ US | 104 |
|  |  |  | Social Connectedness Scale-Revised (+); |  |  |
|  |  |  | Brief Mindfulness Study Social Connectedness Scale (Ø); |  |  |
|  |  |  | Acceptance and Action Questionnaire -2 (+); |  |  |
|  |  |  | Intrapersonal Group^1^: |  |  |
|  |  |  | Social Connectedness Scale-Revised (+); |  |  |
|  |  |  | Brief Mindfulness Study Social Connectedness Scale (Ø); |  |  |
|  |  |  | Acceptance and Action Questionnaire -2 (Ø) |  |  |
| †Carson et al., 2004 | Mindfulness-based relationship enhancement | 8 weeks | Quality of Marriage Index (+); | Healthy couples/ US | 88 (44 couples) |
|  |  |  | Autonomy, Autonomy and Relatedness Inventory (+); |  |  |
|  |  |  | Relatedness, Autonomy and Relatedness Inventory (+); |  |  |
|  |  |  | Inclusion of Other in the Self Scale (+); |  |  |
|  |  |  | Acceptance of Partner Index (+); |  |  |
|  |  |  | Global Distress Scale (+) |  |  |
| †Condon et al., 2013 | Mindfulness/ compassion-based meditation | 8 weeks | Compassionate responding (+) | Healthy adults/ US | 39 |
| Fredrickson et al., 2008 | Loving Kindness Meditation | 7 weeks/ 6 session | Dyadic Adjustment Scale (+); | Healthy adults/ US | 139 |
|  |  |  | Positive relations with others (+) |  |  |
| †Heppner et al., 2008 | Brief mindfulness | 5 min | Aggression: | Undergraduate students/ US | 57 |
|  |  |  | - blast intensity (+); |  |  |
| Hutcherson et al., 2008 | Loving Kindness Meditation Visualisation | 7 min | Explicit: | Healthy adults/ US | 93 (explicit) |
|  |  |  | - target (+); |  | 87 (implicit) |
|  |  |  | - non-target 1 (+); |  |  |
|  |  |  | - non-target 2 (+); |  |  |
|  |  |  | - self (Ø); |  |  |
|  |  |  | - close other (Ø); |  |  |
|  |  |  | - object (+); |  |  |
|  |  |  | Implicit: |  |  |
|  |  |  | - target (+); |  |  |
|  |  |  | - non-target 1 (Ø); |  |  |
|  |  |  | - non-target 2 (Ø); |  |  |
|  |  |  | - self (Ø); |  |  |
|  |  |  | - close other (Ø); |  |  |
|  |  |  | - object (Ø) |  |  |
| †Kang et al., 2013 | Loving Kindness Meditation | 6 weeks | Explicit attitudes | Healthy adults/ US | 101 |
|  |  |  | - homeless (Ø); |  |  |
|  |  |  | - blacks (Ø); |  |  |
|  |  |  | Implicit Association Test (IAT) |  |  |
|  |  |  | - homeless (+); |  |  |
|  |  |  | - blacks (+) |  |  |
| Kemeny et al., 2012 | Meditation/ Emotion regulation training | 8 weeks | Lexical decision measure of compassion (+); | Healthy adults/ US | 76 |
|  |  |  | Micro-Expression Training Tool (+); |  |  |
|  |  |  | Marital Interaction Task (Ø) |  |  |
| † Keng et al., 2012 | Mindfulness Based Stress Reduction | 6 weeks | Spielberger Anger Expression Scale: | Healthy adults/ US | 41 |
|  |  |  | Anger In (+); |  |  |
|  |  |  | Anger Out (+); |  |  |
|  |  |  | Self-Compassion Scale (+) |  |  |
| †Lim et al., 2015 | Mindfulness | 3 weeks | Compassionate responding (+); | Undergraduate students/ US | 56 |
|  |  |  | Emotion Recognition Index (Ø) |  |  |
| †Neff & Germer, 2012 | Mindfulness Compassion Training | 8 weeks | Self-Compassion Scale (+); | Healthy adults/ US | 51 |
|  |  |  | Other-Compassion (+); |  |  |
|  |  |  | Social Connectedness Scale (Ø) |  |  |
| Oman et al., 2009 | 8-week Eight-Point Programme | 8 weeks | Compassionate Love (Ø)^2^; | Healthy adults/ US | 58 |
|  |  |  | Altruism (Ø); |  |  |
|  |  |  | Forgiveness (+)^3^; |  |  |
|  |  |  | Empathy (Interpersonal Reactivity Index): |  |  |
|  |  |  | - empathy perspective taking (Ø); |  |  |
|  |  |  | - emphatic concern (Ø); |  |  |
|  |  |  | - empathy personal distress (Ø) |  |  |
| Oman et al., 2010 | Mindfulness Based Stress Reduction, Faswaran's Eight-Point Programme | 8 weeks | Forgiveness (+)^4^ | Undergraduate students/ US | 44 |
| †Parks et al., 2014 | Loving Kindness Meditation | 8 min | Intergroup Anxiety (+); | Undergraduate students/UK | 78 |
|  |  |  | Attitudes (Ø); |  |  |
|  |  |  | Intentions (+) |  |  |
| †Ramsey & Jones, 2015 (study 2) | Brief mindfulness | 5 min | Aggression: | Undergraduate students/ US | 100 |
|  |  |  | - team picking (Ø); |  |  |
|  |  |  | - ball tossing (+) |  |  |
| †Robins et al., 2012 | Mindfulness Based Stress Reduction | 8 weeks | Spielberger Anger Expression Scale: | Healthy adults/ US | 41 |
|  |  |  | Anger In (+); |  |  |
|  |  |  | Anger Out (+); |  |  |
|  |  |  | Self-Compassion Scale (+) |  |  |
| †Rosenberg et al., 2015 | Meditation | 3 months | Overall emotion - facial behaviour (Ø); | Healthy adults | 56 |
|  |  |  | Rejection behaviours – facial expression (+) |  |  |
| †Smeets et al., 2014 | Mindfulness Compassion Training | 3 weeks | Self-Compassion Scale short form (+); | Undergraduate students/ Netherlands | 49 |
|  |  |  | Social Connectedness Scale-Revised (+) |  |  |
| †Tan et al., 2014 | Mindfulness | 5 min | Cyber Ball Social Exclusion Game (+); | Healthy adults/ Hong Kong | 72 |
|  |  |  | Reading the Mind in the Eyes Test (+) |  |  |
| †Wallmark et al., 2013 | Loving Kindness Meditation | 8 weeks | Self-Compassion Scale (+); | Healthy adults/ Sweden | 42 |
|  |  |  | Empathy (Interpersonal Reactivity Index): |  |  |
|  |  |  | - empathy perspective taking (+); |  |  |
|  |  |  | - empathy personal distress (Ø); |  |  |
|  |  |  | - emphatic concern (Ø) |  |  |
|  |  |  | - altruistic orientation (Ø) |  |  |
| Yusainy & Lawrence, 2015 | Brief mindfulness | 15 min | Aggression | Undergraduate students/ UK | 110 |
|  |  |  | - blast intensity (+); |  |  |
|  |  |  | - handgrip task (+) |  |  |
| *Note*: † Included in the meta-analysis; Ø no effect (within ±5%); + improved statistically; US United States, UK United Kingdom | | | | | |
| 1 Comparisons are against the control group. | | | | | |
| 2 Compassionate Love was measured with two items from the Daily Spiritual Experiences Scale. | | | | | |
| 3 Forgiveness was measured with two items from the forgiveness short form of the Multidimensional Measure of Religiousness/Spirituality. | | | | | |
| 4 Forgiveness was measured with a 6-item subscale of the Heartland Forgiveness Scale. | | | | | |

| Supplementary Table 2 | | | | | | | | |
| --- | --- | --- | --- | --- | --- | --- | --- | --- |
| *Quality Assessment* | |  |  |  |  |  |  |  |
| Source | Random sequence generation | Advertised as meditation study | Confound variables (e.g. demand characteristics) | Blinding of outcome assessment | Selective reporting | Intervention Teacher* | Type of control group | Overall grade (1 = strong, 2=moderate 3=weak) |
| Arch et al., 2014 | Not reported | No | Yes | No | No | Audio recording | Active & Passive | 2 |
| Bowen et al., 2012 | Not reported | Yes | No | No | No | Third & fourth author | Active | 2 |
| Carson et al., 2004 | Not reported | Yes | No | No | Yes | Two first authors | Passive | 3 |
| Condon et al., 2013 | Not reported | Yes | No | No | No | Second to last author | Passive | 3 |
| Frederickson et al., 2008 | Not reported | Yes | No | No | Yes | Last author | Passive | 3 |
| Heppner et al., 2008 | Not reported | No | No | No | No | Audio recording | Passive | 3 |
| Hutcherson et al., 2008 | Not reported | Yes | No | No | Yes | Paper instructions | Passive | 3 |
| Kang et al., 2013 | Not reported | Yes | No | No | Yes | External instructor | Active & Passive | 3 |
| Keng et al., 2012 | Not reported | Yes | Yes | No | No | Last author | Passive | 3 |
| Kemeny et al., 2012 | Yes | Yes | No | No | Yes | Middle and last author | Passive | 3 |
| Lim et al., 2015 | Not reported | No | No | No | No | Mobile application | Active | 2 |
| Neff & Germer, 2012 | Not reported | Yes | No | No | Yes | Two clinical psychologists | Passive | 3 |
| Oman et al., 2009 | Not reported | Yes | No | No | Yes | Information could not be obtained | Passive | 3 |
| Oman et al., 2010 | Not reported | Yes | No | No | Yes | Information could not be obtained | Passive | 3 |
| Parks et al., 2014 | Not reported | No | No | No | Yes | Audio recording | Passive | 3 |
| Ramsey & Jones, 2015 | Not reported | No | No | No | No | Audio recording | Active | 2 |
| Robins et al., 2012 | Not reported | Yes | Yes | No | No | Last author | Passive | 2 |
| Rosenberg et al., 2015 | Stratified | Yes | No | Partly | No | Second to last author | Passive | 3 |
| Smeets et al., 2014 | Yes | No | No | No | Yes | First author & external instructor | Active | 3 |
| Tan et al., 2014 | Not reported | No | No | Yes | No | Audio recording | Active | 2 |
| Wallmark et al., 2013 | Yes | Yes | No | No | Yes | First two authors | Passive | 3 |
| Yusainy & Lawrence, 2015 | Yes | No | No | No | No | Audio recording | Active | 2 |
| *Note:* *Authors were emailed where information about the meditation teacher was not available in the published report. | | | | | | | | |
